# Proteomic based approach for characterizing 4-hydroxy-2-nonenal induced oxidation of buffalo (*Bubalus bubalis*) and goat (*Capra hircus*) meat myoglobins

**DOI:** 10.1186/s12953-016-0108-7

**Published:** 2016-11-16

**Authors:** Naveena B. Maheswarappa, K. Usha Rani, Y. Praveen Kumar, Vinayak V. Kulkarni, Srikanth Rapole

**Affiliations:** 1 National Research Centre on Meat, Chengicherla, Hyderabad, Telangana 500092 India; 2 Proteomics Lab, National Centre for Cell Science, Pune, 411007 India

**Keywords:** Myoglobin-HNE adduction, Redox instability, OFFGEL electrophoresis, 2-Dimensional gel electrophoresis, Mass spectrometry

## Abstract

**Background:**

Myoglobin (Mb) is a sarcoplasmic heme protein primarily responsible for meat color and its chemistry is species specific. 4-hydroxy-2-nonenal (HNE) is a cytotoxic lipid derived aldehyde detected in meat and was reported to covalently adduct with nucleophilic histidine residues of Mb and predispose it to greater oxidation. However, no literature is available on characterization of lipid oxidation induced oxidation of Indian water buffalo (*Bubalus bubalis*) and goat (*Capra hircus*) myoglobins.

**Methods:**

Present study characterize the Mb extracted from water buffalo and goat cardiac muscles using two-dimensional gel electrophoresis (2DE), OFFGEL electrophoresis and mass spectrometry (MS). Purified buffalo and goat bright red oxymyoglobin were reacted with HNE in-vitro at physiological pH (7.4) and temperature (37 °C) conditions and the formation of oxidised brown metmyoglobin was measured. The Mb-HNE adducts were detected using MALDI-TOF MS, whereas specific sites of adduction was determined using ESI-QTOF MS/MS.

**Results:**

Purified buffalo and goat Mb samples revealed a molecular mass of 17,043.6 and 16,899.9 Daltons, respectively. The 2DE analysis exhibited 65 (sarcoplasmic protein extract) and 6 (pure Mb) differentially expressed (*P* < 0.05) protein spots between buffalo and goat samples. OFFGEL electrophoresis revealed an isoelectric point of 6.77 and 7.35 respectively, for buffalo and goat Mb’s. In-vitro incubation of HNE with bright red buffalo and goat oxymyoglobin’s at pH 7.4 and 37 °C resulted in pronounced (*P* < 0.05) oxidation and formation of brown metmyoglobin. MALDI-TOF MS analysis of Mb-HNE reaction mix revealed covalent binding (via Michael addition) of 3 and 5 molecules of HNE with buffalo and goat Oxy-Mb’s, respectively. ESI-QTOF MS/MS identified seven and nine histidine (HIS) residues of Mb that were readily adducted by HNE in buffalo and goat, respectively.

**Conclusion:**

The study demonstrated better redox stability of buffalo Mb than goat Mb. Our findings confirm the hypothesis that relative effect of HNE was greater for Mb’s with 12 ± 1 HIS residues than Mb’s with 9 HIS residues and helps meat processors in developing species-specific processing strategies to reduce the color variability.

## Background

India is the largest producer and exporter of water buffalo (*Bubalus bubalis*) meat in the world and accounts for 23.5% of global bovine meat exports [1]. Indian water buffalo meat is considered lean with higher protein and ash content relative to cattle meat (beef) [2]. Owing to its healthier properties, buffalo meat is emerging as an important red meat source in several Middle-east, South-east and European countries. India is the second largest producer of goat (*Capra hircus*) meat next to China. Goat meat is a popular red meat in many regions of the world and the consumer preference of goat meat is almost universal depending on cultural traditions and social and economic conditions [3]. Buffalo meat is darker compared to beef and the darkness is attributed to higher myoglobin (Mb) content (5.0 mg/g) [4]. Dosi et al. (2006) have studied the primary structure of buffalo Mb using a combined approach of Edman degradation and MALD-TOF mass spectrometry and found a difference of three amino acids out of 153 compared to beef Mb [5]. These authors have also studied the stability, autoxidation and percent metmyoglobin formation in beef and buffalo Mb’s and reported identical results between them. Another important livestock species, goat was reported to share 98.7% sequence similarity with sheep than with buffalo which has got 95.4% sequence similarity. Goat meat was reported to be darker, more red and have higher sarcoplasmic protein content than sheep meat [6]. Primary structure of goat Mb was determined by Suman et al. (2009) who reported that goat Mb shared 98.7% similarity with sheep Mb and the distal (64) and proximal (93) histidines responsible for coordinating the heme group and reversible binding of oxygen are conserved in goat Mb, similar to other meat-producing livestock [7].

Molecular properties and overall structure of the Mb protein from different species have been reported to be very similar although there are differences in the amino acid composition and the exact length of the polypeptide chain. Primary structure of Mb influences meat color stability via mechanisms such as autoxidation, heme retention, structural stability, thermostability, and oxygen affinity [8]. Oxidation of ferrous oxymyolobin (Oxy-Mb) to ferric metmyoglobin (Met-Mb) will result in conversion of desirable bright red color of the meat into undesirable brown color [9]. Several researchers have demonstrated that myoglobin oxidation and lipid oxidation are interrelated and the acceleration of one will exacerbate the other [9–12]. These researchers have demonstrated acceleration of heme protein oxidation in the presence of unsaturated aldehydes generated by free radical-induced lipid peroxidation of polyunsaturated fatty acids. 4-Hydroxy-2-nonenal (HNE) is an α, β-unsaturated aldehyde derived from the oxidation of ω-6 polyunsaturated fatty acids and has been reported in meat [13]. It was reported to involve in the onset and progression of many pathological conditions such as cardiovascular and neurodegenerative diseases due to its ability to react with the nucleophilic sites of proteins and peptides to form covalently modified biomolecules [14]. Because of high reactivity of HNE with proteins, researchers have concluded the possibility of using HNE-adducted proteins as markers for oxidative damage [15]. HNE was also reported to induce redox instability of Mb’s from horse, cattle, pig, sheep, deer, chicken, turkey, emu and ostrich meats under different conditions and in all cases Oxy-Mb oxidation was promoted [9, 10, 12, 16–18]. These studies suggest that, HNE accelerates Oxy-Mb oxidation in-vitro by covalent modification at histidine residues. Using LC-MS/MS analysis Alderton et al. (2003) identified six histidine residues of beef Mb that were readily adducted by HNE, including the proximal (HIS 93) and distal (HIS 64) histidines associated with the heme group at pH 7.4 and 37 °C [10]. However, Suman et al. (2007) demonstrated the adduction of HNE with three and seven histidine residues in porcine and beef Mb’s under similar conditions [12]. These authors have hypothesized that, the lesser number of nucleophilic HIS residues and their specific locations in porcine Mb are responsible for the greater redox stability of porcine Mb in the presence of lipid oxidation products relative to beef. They further concluded that, preferential HNE adduction at proximal HIS 93 was observed only in bovine Oxy-Mb and hence lipid oxidation induced Oxy-Mb oxidation was potentially more critical in beef than pork. Yin et al. (2011) studied the HNE induced oxidation of Oxy-Mb’s from seven different meat animals and concluded that, the relative effect of HNE was greater for Mb’s that contained 12 ± 1 HIS residues than for those that contained nine HIS residues [18]. All these researchers suggested a species specific effect of HNE on Mb. In the present study we propose to characterize the Mb-HNE adductions in two ruminant species viz, water buffalo and goat with 13 and 12 HIS residues in their Mb, respectively.

Almost all the studies related to meat color have used liquid chromatography-mass spectrometry (LC-MS) based tools to characterize the primary structure of Mb, to understand the lipid oxidation induced oxidation of Oxy-Mb and to determine the redox instability of mutant sperm whale Mb [5, 9, 10, 19]. Investigations have documented the contribution of sarcoplasmic proteome on muscle-specificity in beef color [20, 21]. However, to our knowledge no studies have been reported on use of two-dimensional gel electrophoresis (2DE) coupled with mass spectrometry to characterize purified Mb’s especially from water buffalo and goat meats. Further, to our knowledge HNE induced oxidation of buffalo and goat Mb’s and identification of specific amino acids with which it reacts was not previously reported. Therefore, our objectives were 1) to characterize the Mb using 2DE, OFFGEL electrophoresis and mass spectrometry from two important emerging meat animals, buffalo and goat and 2) to investigate the potential binding of HNE to buffalo and goat meat Mb’s and determine how it affects redox stability under different temperature and pH conditions. The present study is the first to characterize the livestock Mb’s using 2DE and OFFGEL electrophoresis.

## Results and discussion

### Purification of buffalo and goat Mb’s

**Fig. 1:**
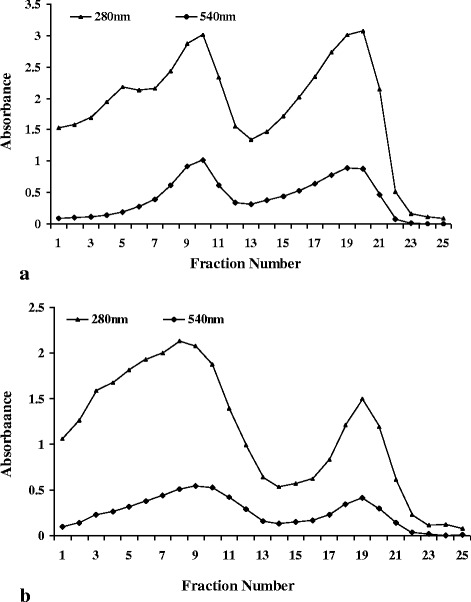
Elution profile of (**a**) buffalo and **b** goat cardiac myoglobin from Sephacryl S-200 HR gel filtration chromatography. The column was equilibrated and eluted with 5 mM Tris–HCl + 1 mM EDTA buffer, pH 8.0 at a flow rate of 0.1 mL/min

Isolation and purification of beef, pork, sheep, turkey and chicken myoglobins (Mbs) either from skeletal, heart or smooth muscles using ammonium sulfate precipitation and gel filtration has been reported by different authors [7, 12, 16, 18]. The extraction and purification of Mb from buffalo and goat is minimally investigated and to our knowledge only two papers are available in the literature for buffalo and goat Mb extraction and characterization [5, 7]. Dosi et al. (2006) have extracted buffalo Mb in Milli Q water and further purified through dialysis, Sephacryl S-100 column chromatography followed by Diethylaminoethyl (DEAE) anion exchange chromatography [5]. Goat Mb was extracted and purified by Suman et al. (2009) using the modified procedure of Faustman and Phillips (2001) [7, 22]. These authors have used 50% ammonium sulfate saturation in place of 70% as originally suggested by Faustman and Phillips (2001) [22]. In the present study, we have successfully extracted and purified both buffalo and goat Mb’s as per the procedure suggested by Faustman and Phillips (2001) with 70% ammonium sulfate precipitation and gel filtration chromatography using Sephacryl S-200 HR [22]. Purification of ammonium sulfate precipitated and dialyzed Mb on Sephacryl S-200 HR gel-filtration column revealed two major peaks at A_540_ for buffalo (Fig. 1a) and goat (Fig. 1b). This elution pattern suggests clear separation of hemoglobin and Mb on gel filtration column. Hemoglobin (~64 kDa) elutes first followed by Mb (~17 kDa) and these findings confirm with our earlier reports for chicken and turkey Mb’s [16].

### SDS-PAGE and two-dimensional gel electrophoresis (2DE) of buffalo and goat Mb’s

**Fig. 2:**
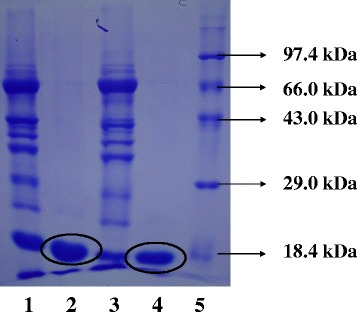
SDS-PAGE pattern of samples obtained during purification of buffalo and goat cardiac myoglobins. Lane 1, buffalo sarcoplasmic protein extract; Lane 2, pure buffalo myoglobin; Lane 3, goat sarcoplasmic protein extract; lane 4, pure goat myoglobin; lane 5, molecular weight standard

**Fig. 3:**
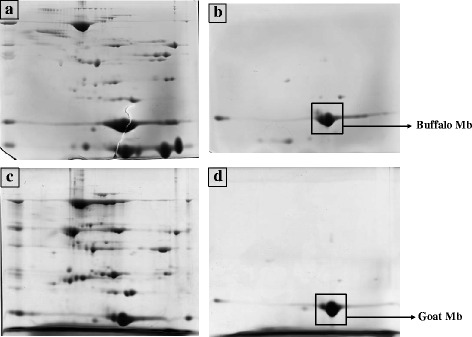
Coomassie-stained two-dimensional gel of (**a**) buffalo sarcoplasmic protein extract, **b** purified buffalo myoglobin, **c** goat sarcoplasmic protein extract and (**d**) purified goat myoglobin

The SDS-PAGE of pooled fractions from second peak which is supposed to be Mb, consistently revealed the presence of single band at approximately 17 kDa level in both buffalo and goat (Fig. 2) samples. Dosi et al. (2006) also reported a single protein band for purified buffalo Mb on SDS-PAGE without any detectable contaminating protein bands [5]. Our findings confirm that buffalo and goat Mb’s can be purified using different ammonium sulphate precipitation, dialysis, filtration and gel filtration chromatography steps as suggested by Faustman and Phillips (2001) [22]. To further check the purity of Mb, 2DE of sarcplasmic extract and the purified Mb’s from buffalo and goat was carried out. Wu et al. (2016) used 2DE and tandem MS to differentiate sarcoplasmic proteome of *Longissimus lumborum* (LL) and *Psoas major* (PM) muscles of Chinese *Luxi* yellow cattle and identified the proteins mainly involved in glycolytic metabolism which contributed to better meat color stability in LL compared to PM [23]. In the present study, sarcoplasmic proteome extract and gel-filtered purified Mb from buffalo and goat were characterized using 2DE. The 2DE was done to separate differentially expressed sarcoplasmic proteins mainly consist of myoglobin, hemoglobin, cytochrome and wide variety of endogenous enzymes that are associated with meat color. The analysis of 2DE gels revealed separation of 508 and 563 spots respectively, in buffalo and goat sarcoplasmic extracts (Fig. 3a and c). The class analysis table by analysis of variance (ANOVA) of buffalo and goat sample gels indicated 65 differential spots (*p* < 0.05) which had protein spot expression of 1.5 fold or more between them. Variation in spot intensity/abundance was consistently observed across three gels each for buffalo and goat samples. Joseph et al. (2012) observed a total of 180 protein spots for sarcoplasmic protein extract from beef *Longissimus lumborum* and indicated 17 differentially abundant protein spots in comparison to *Psoas major* muscle [21]. Purified Mb’s revealed 19 and 20 spots, respectively for buffalo (Fig. 3b) and goat (Fig. 3d) samples with six spots being differentially expressed (*p* < 0.05) between them. The over-abundance of spots in goat sarcoplasmic proteins might be due to post-translational modification of some proteins at a greater degree in goat than in buffalo which may compromise color stability. Sarcoplasmic proteome governs different biochemical processes influencing meat color stability and their interactions with myoglobin are critical to meat color. Present study established the differential abundance of sarcoplasmic proteome between buffalo meat and goat meats. A beef color stability variation due to differences in sarcoplasmic proteome was reported by Joseph et al. (2012) [21]. Differential analysis of sarcoplasmic proteome using image analyses of the Coomassie-stained 2DE gels was reported to contain twelve differentially abundant protein spots in color-stable and color-labile beef longissimus muscles [20]. Our findings suggest significant variation in sarcoplasmic proteome between buffalo and goat samples. Eventhough, purification of buffalo and goat Mb’s using gel-filtration resulted in single band at 17 kDa in SDS-PAGE anlaysis, the 2DE revealed few other contaminating proteins with very little intensity/abundance. However, in both buffalo and goat 2DE gels, single large Mb fraction was observed at same location. This spot was further analysed using peptide mass fingerprinting (PMF) for confirmation of Mb.

### Determination of molecular mass and peptide mass fingerprinting using MALDI-TOF MS

**Fig. 4:**
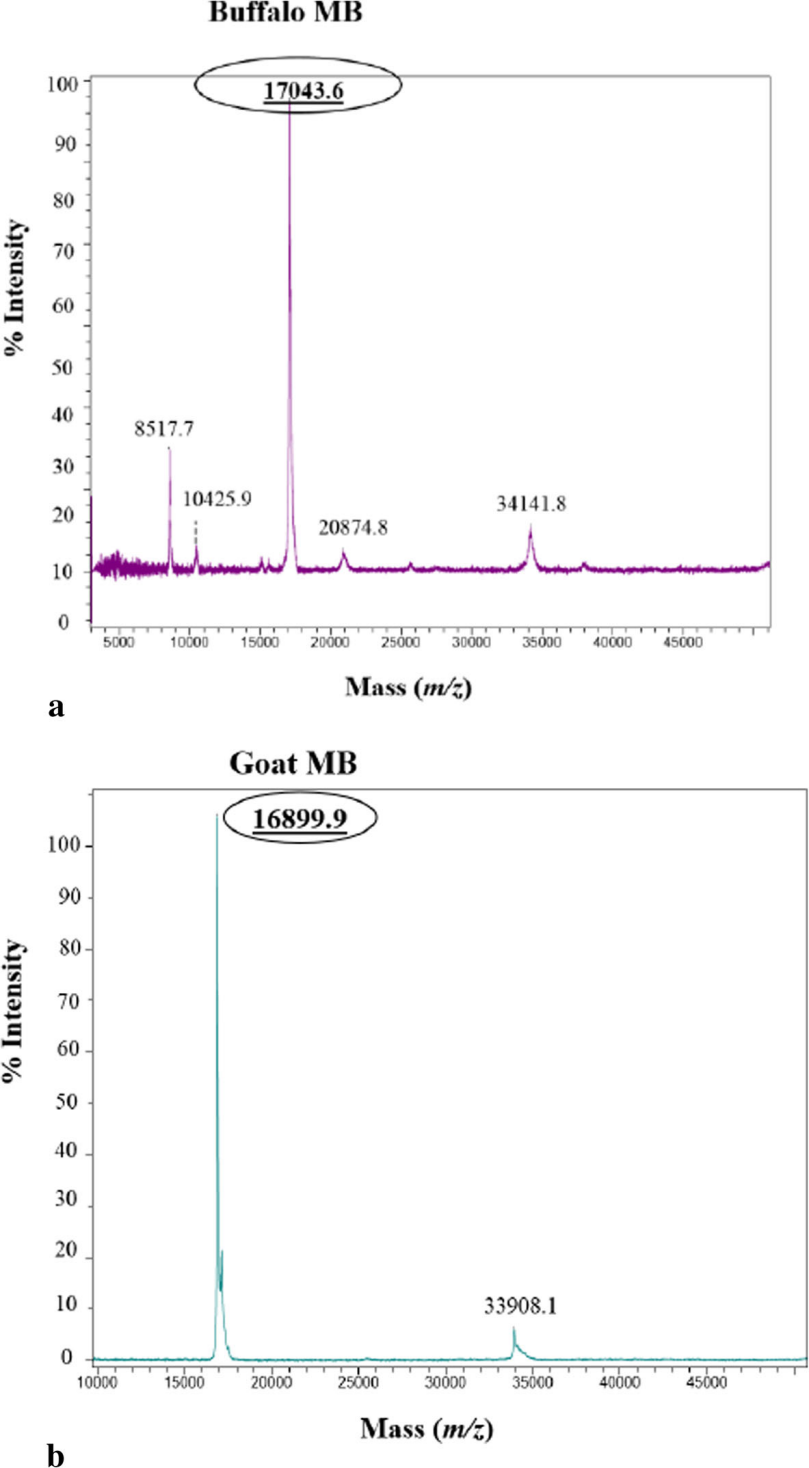
MALDI-TOF mass spectra of purified (**a**) buffalo and **b** goat myoglobins

Mass spectrometry analysis was performed only for the purified Mb’s to determine molecular mass and to confirm their identity through PMF. The MALDI-TOF MS analysis of intact buffalo Mb revealed the mass of 17043.6 Daltons (Fig. 4a) which is 97.6 Daltons more than beef Mb [24]. The MS analysis of goat Mb revealed a mass of 16899 Daltons (Fig. 4b) which is 24 Daltons less than sheep Mb [25]. The observed molecular mass for buffalo Mb in the present study is 9.6 Daltons higher than the report of Dosi et al. (2006) for Italian water buffalo Mb extracted from skeletal muscles [5]. The molecular mass of goat Mb observed in the present study is 75 Daltons higher than the report of Suman et al. (2009) [7]. In general it is agreed that the mass of Mb for all the livestock and poultry species is around 17000 Daltons.

Purified Mb protein separated by 2DE gel was identified by MALDI-TOF MS. The analysis of generated peptides along with their *m*/*z* values and intensities revealed an abundant peptide with a *m*/*z* value of 748 for buffalo Mb compared to goat Mb in which a peptide with *m*/*z* of 1592 was most abundant (data not shown). The PMF results with details of molecular weight search score, number of matched peptides, nominal and observed mass along with % sequence coverage has been given in Table 1. Clear distinction can be made from the result where there is a reducing ion score for goat samples relative to buffalo samples. Peptide masses detected in the present study were matched for buffalo and goat Mb proteins in a database which showed the sequence coverage of 39.61% for buffalo and 41.55% for goat. Four peptides matched for buffalo Mb, whereas for goat Mb six peptides were matched. Furthermore, MALDI-TOF analysis consistently gave information on the peptide-mass fingerprint from position 2–119 for buffalo and 2–153 for goat samples. These results confirmed the identity of purified protein spot on 2DE gel as buffalo and goat Mb’s. The observed differences in peptides and their masses between buffalo and goat samples is because of the fact that buffalo Mb has 95.4% sequence similarity with goat Mb (expacy.com).

**Table 1:** Results of peptide mass fingerprinting (PMF) for identification of water buffalo (*Bubalus bubalis*) and goat meat (*Capra hircus*) myoglobins

Species	Accession No.	Score	No. matched peptides	Nominal mass	Observed mass	% Sequence coverage highlighting the matched peptides
Buffalo (*Bubalusbubalis*)	giI116248552	289	4	17155.0	17043.6	Sequence coverage = 39.61% M**GLSDGEWQLVLNAWGKVETDVAGHGQEVL IR**LFTGHPET LEKFDKFKHL KTEAEMKASE DLKK**HGNTVLTALGGILK**KK GHHEAEVKHL AESHANKHKI PVK**YLEFISDAIIHVLHDK**H PSDFGADAQA AMSKALELFR NEMAAQYKVL GFHG
Goat (*Capra hircus*)	giI118595750	79	6	17043.0	16899.9	Sequence coverage = 41.55% M**GLSDGEWTLVLNAWGKVEADVAGHGQEVLIR**LFTGHPET LEKFDKFKHL KTGAEMKASE DLKKH**GNTVLTALGGILK**KK GHHEAEVKHL AESHANKHKI PVKYLEFISD AIIHVLHAKH PSDFGADAQG AMSK**ALELFRNDMAAQYKVL GFQG**

### OFFGEL electrophoresis

For the first time, we have attempted to use the OFFGEL electrophoresis to determine the isoelectric point (p*I*) of buffalo and goat Mb’s. OFFGEL electrophoresis uses multi-compartment chamber on top of an IPG strip which provides the desired pH gradient and solubilized proteins migrate through the strip until they reach their p*I* at a given compartment and then return into solution [26]. Using the Agilent 3100 OFFGEL fractionator high resolution mode (24 fractions per 24 cm, 3–10 IPG strip) with the Agilent’s proprietary starter kit, buffalo and goat Mb’s were fractionated separately. The p*I* was calculated by dividing the seven pH units of 3–10 gradient by the number of compartments, which is 24 in the present experiment. Each compartment corresponds to a pH resolution of 0.29. After completion of OFFGEL electrophoresis, the fractionated samples from each well, in solution form were taken out and subjected to SDS-PAGE. Out of 24 collected fractions each for buffalo and goat Mb’s, single clear band corresponding to 17 kDa molecular mass was obtained (figure not shown) at 14 and 16th fractions, respectively for buffalo and goat. Based on the fraction number corresponding to 17 kDa band, the p*I* of buffalo and goat Mb’s was calculated to be 6.77 and 7.35, respectively. Post-translational modification of proteins via phosphorylation leads to an acidic shift in the isoelectric pH which may result in variation [27]. The p*I* values investigated in the present study are almost similar to theoretical p*I* values of 6.71 and 7.38 for buffalo and goat Mb’s respectively (web.expasy.org).

### HNE induced oxidation of buffalo and goat Oxy-Mb’s

**Fig. 5:**
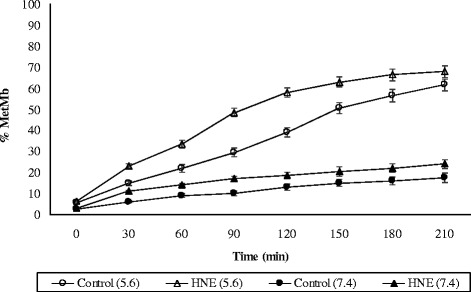
Oxidation of buffalo meat oxymyoglobin reacted with HNE (treatment) and without HNE (control) at 25 °C, pH 5.6 and 37 °C, pH 7.4. Standard error bars are indicated (*n* = 3)

**Fig. 6:**
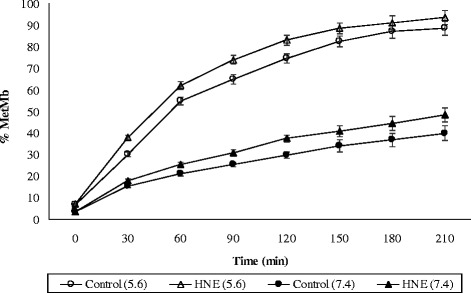
Oxidation of goat meat oxymyoglobin reacted with HNE (treatment) and without HNE (control) at 25 °C, pH 5.6 and 37 °C, pH 7.4. Standard error bars are indicated (*n* = 3)

HNE is a well-documented secondary product of ω-6 polyunsaturated fatty acids oxidation. Various researchers have utilized HNE as a model oxidation product to study its interaction with Oxy-Mb from different livestock and poultry species as mentioned earlier [28]. To understand the proxidative effect of α,β-unsaturated aldehyde, HNE was incubated with buffalo and goat Oxy-Mb’s for 3.5 h at 25 °C, pH 5.6 and 37 °C, pH 7.4 to represent the pH values of post-mortem muscle and physiological conditions, respectively. Pure Mb without HNE (control) was also incubated along with HNE reacted Mb (treatment) for comparison under similar conditions. The %Met-Mb formed during incubation of buffalo and goat Oxy-Mb’s with HNE (treatment) and without HNE (control) at 25 °C, pH 5.6 and 37 °C, pH 7.4 are presented in Figs. 5 and 6, respectively. When HNE was reacted with Oxy-Mb solutions, the %Met-Mb formation was greater (*p* < 0.05) than controls at all the temperatures and pH conditions for both buffalo and goat. As expected, the %Met-Mb formation is higher (*p* < 0.05) at 37 °C compared to 25 °C. Oxy-Mb oxidation of both buffalo and goats were higher (*p* < 0.05) at pH 5.6 relative to pH 7.4, a result attributed to greater autoxidation. Similar results were observed during the incubation of HNE with equine, bovine and porcine Oxy-Mb’s [9, 10, 12]. In general, the oxidising effect of HNE is greater at 7.4 compared to 5.6 potentially because of decreased nucleophilicity of candidate histidine residues at this pH [29]. Oxymyoglobin-HNE adduct formation was greater at pH 7.4 than at pH 5.6, because at acidic pH several ionizable imidazole groups (in HIS) are protonated and therefore, less reactive with HNE. The pKa value for the imidazole group of histidine is approximately 6.0, and therefore HIS residues would act as suitable nucleophilic targets for aldehyde adduction at pH 7.4. However, protonation of the imidazole ring at pH 5.6 would render HIS residues a less favorable candidate for aldehyde adduction via Michael addition. This will contribute to the greater number of HNE adduction at pH 7.4 than at typical meat pH (5.6) [12]. Further, Oxy-Mb oxidation at pH 5.6 will progress significantly rapid enough to mask any observable redox destabilizing effect of HNE. Considering these reasons and the earlier studies we used pH 7.4 condition for further characterization of Mb-HNE adduction.

**Fig. 7:**
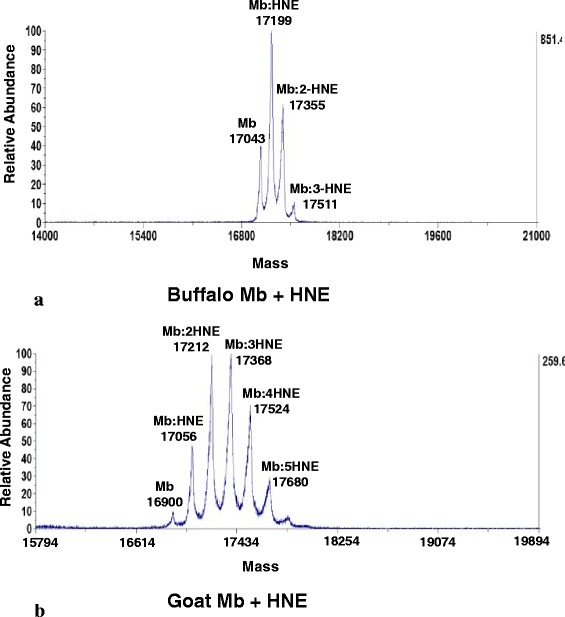
MALDI-TOF MS spectrum of (**a**) buffalo meat oxymyoglobin (0.15 mM) reacted with HNE (1.0 mM) and **b** goat meat oxymyoglobin (0.075 mM) reacted with HNE (0.5 mM) at pH 7.4 and 37 °C for 3.5 h

MALDI-TOF MS analysis of Mb-HNE reaction mix (intact protein) at the end of incubation period (after 3.5 h) at pH 7.4 and 37 °C revealed covalent binding of upto three and five molecules of HNE to buffalo (Fig. 7a) and goat (Fig. 7b) Oxy-Mb’s, respectively. These results indicate that HNE adducts were formed via Michael addition as the adduct peaks corresponded to the mass of myoglobin plus 156 Da, the molecular mass of HNE. These results suggest that buffalo myoglobin was less susceptible to nucleophilic attack and subsequent adduction with HNE, compared to goat Mb. This explains the reason for higher %Met-Mb formation observed for goat Oxy-Mb than buffalo Oxy-Mb when incubated with HNE. Research to date has demonstrated that a multitude of factors (endogenous and exogenous) contribute to meat color stability and biochemistry. Among several factors, adduction of lipid derived aldehydes with Oxy-Mb was reported to cause increased %Met-Mb formation and redox instability in different livestock and poultry species [8]. Michael adduction of HNE with apomyoglobin (model heme protein lacking cysteine residue) was confirmed by Bolgar and Gaskell (1996) using ESI-MS and indicated three to 10 HNE adducts per protein molecule [30]. Faustman et al. (1999) and Alderton et al. (2003) have reported tri-adducts of HNE with equine and beef Oxy-Mb’s incubated at pH 7.4 and 37 °C after 3 hours of incubation [9, 10]. Species specific effect of Mb-HNE adducts were observed by previous researchers as indicated by mono-adducts in porcine Mb at pH 5.6 [12], di-adducts for chicken and turkey at pH 7.4 [16], mono-adducts for emu and ostrich at pH 7.4 [17] during incubation for different time periods. Our present findings synergistically add to the existing knowledge that lipid oxidation induced oxidation of myoglobin from red meat species (equine meat, beef and sheep meat) is more pronounced relative to white meats (poultry species).

Adduction of Oxy-Mb with 4-hydroxy-2-nonenal would be expected to alter the protein’s tertiary structure and predispose it to greater oxidation [14]. The specific sites of HNE adduction in buffalo and goat Mb’s incubated with and without (control) HNE were further investigated using ESI-QTOF MS/MS specifically at histidine (HIS) site as the earlier researchers have confirmed that HIS was the sole amino acid adducted by HNE [10, 12]. The spectra corresponding to unadducted and adducted Mb peptides were identified and the *b*- and *y*-series ions for seven buffalo Mb peptides with HNE adductions are summarized in Table 2. In the present study, we identified seven nucleophilic histidine residues for buffalo Mb confirmed to be adducted with HNE. The HIS 24, 36, 81, 88, 93, 119 and 152 each had a mass addition of 158 Da which is 2-Da higher than the mass of HNE. Previous study by Alderton et al. (2003) revealed a mass increment of 156 Da due to HNE adduction via Michael addition [10]. The difference of 2-Da (156 vs. 158) in the present study could be due to protonation of HNE-adducted HIS which might have resulted in a 2-Da addition to the adduct’s molecular mass and it is in agreement with Suman et al. (2007) [12]. For goat Mb we identified nine nucleophilic histidine residues confirmed to be adducted with HNE. The HIS 24, 36, 64, 81, 88, 97, 113, 116 and 119 each had a mass addition of 158 Da. The spectra corresponding to unadducted and adducted Mb peptides were identified and the *b*- and *y*-series ions for goat Mb peptides with HNE adductions are summarized in Table 3. Compared to buffalo Mb, goat Mb had two additional HNE adductions. Our results for buffalo Mb-HNE adductions were similar to the findings of Suman et al. (2007) who has also observed seven beef Mb-HNE adductions under similar pH and temperature conditions [12]. This is because of the fact that both buffalo and cattle shares 98% sequence similarity with 13 total histidine residues in each located at the same position. Similar to the findings of Suman et al. (2007), HIS 88 and HIS 93 originating from the same peptide (HLAESHANK, position 88–96) were found adducted to HNE [12]. Simultaneous adduction at histidine 88 and histidine 93 was not detected because of the fact that alkylation of one histidine prevents the other nearby histidine residue from being alkylated due to steric hindrance [19].

**Table 2:** MS/MS spectral features of unadducted and HNE-adducted water buffalo (*Bubalus bubalis*) myoglobin peptides

Peptide position^a)^	Peptide sequence^b)^	Modification and mass shift	Precursor *m*/*z*	b and y ions identified^c)^
17–31	VETDVAGHGQEVLIR	Unadducted	1604.83	b ions: 100.08 (b1), 229.12 (b2), 300.16 (b3), 415.18 (b4), 514.25 (b5), 585.29 (b6), 642.31 (b7), 779.37 (b8), 836.39 (b9), 964.45 (b10), 1093.49 (b11), 1192.56 (b12), 1305.64 (b13), 1418.73 (b14) y ions: 173.10 (y1), 286.19 (y2), 399.27 (y3), 498.34 (y4), 627.38 (y5), 755.44 (y6), 812.46 (y7), 949.52 (y8), 1006.54 (y9), 1077.58 (y10), 1176.65 (y11), 1291.68 (y12), 1362.71 (y13), 1491.76 (y14)
17–31	VETDVAG**H**GQEVLIR	HNE + 158.1	1604.84	b ions: 100.08 (b1), 229.12 (b2), 300.16 (b3), 415.18 (b4), 514.25 (b5), 585.29 (b6), 642.31 (b7), **937.50** (**b8**), **994.52** (**b9**), **1122.58** (**b10**), **1251.62** (**b11**), **1350.69** (**b12**), **1463.77** (**b13**), **1576.86** (**b14**) y ions: 173.10 (y1), 286.19 (y2), 399.27 (y3), 498.34 (y4), 627.38 (y5), 755.44 (y6), 812.46 (y7), **1107.65** (**y8**), **1164.67** (**y9**), **1235.71** (**y10**), **1334.78** (**y11**), **1449.81** (**y12**), **1520.84** (**y13**), **1649.89** (**y14**)
32–42	LFTGHPETLEK	Unadducted	1271.66	b ions: 114.09 (b1), 261.16 (b2), 362.21 (b3), 419.23 (b4), 556.29 (b5), 653.34 (b6), 782.38 (b7), 883.43 (b8), 996.52 (b9), 1125.56 (b10) y ions: 145.10 (y1), 274.14 (y2), 387.22 (y3), 488.27 (y4), 617.31 (y5), 714.37 (y6), 851.43 (y7), 908.45 (y8), 1009.50 (y9), 1156.56 (y10)
32–42	LFTG**H**PETLEK	HNE + 158.1	1253.65	b ions: 114.09 (b1), 261.16 (b2), 362.21 (b3), 419.23 (b4), **714.42** (**b5**), **811.47** (**b6**), **940.51** (**b7**), **1041.56** (**b8**), **1154.65** (**b9**), **1283.69** (**b10**) y ions: 145.10 (y1), 274.14 (y2), 387.22 (y3), 488.27 (y4), 617.31 (y5), 714.37 (y6), **1009.56** (**y7**), **1066.58** (**y8**), **1167.63** (**y9**), **1314.69** (**y10**)
80–87	GHHEAEVK	Unadducted	1893.90	b ions: 58.03 (b1), 195.09 (b2), 332.15 (b3), 461.19 (b4), 532.23 (b5), 661.27 (b6), 760.34 (b7) y ions: 145.10 (y1), 244.17 (y2), 373.21 (y3), 444.25 (y4), 573.29 (y5), 710.35 (y6), 847.41 (y7)
80–87	G**H**HEAEVK	HNE + 158.1	1893.93	b ions: 58.03 (b1), **353.22** (**b2**), **490.28** (**b3**), **619.32** (**b4**), **690.36** (**b5**), **819.40** (**b6**), **918.47** (**b7**) y ions: 145.10 (y1), 244.17 (y2), 373.21 (y3), 444.5 (y4), 573.29 (y5),710.35 (y6), **1005.54** (**y7**)
88–96	HLAESHANK	Unadducted	1253.65	b ions: 138.07 (b1), 251.15 (b2), 322.19 (b3), 451.23 (b4), 538.26 (b5), 675.32 (b6), 746.36 (b7), 860.40 (b8) y ions: 145.10 (y1), 259.14 (y2), 330.18 (y3), 467.24 (y4), 554.27 (y5), 683.70 (y6), 754.78 (y7), 867.94 (y8)
88–96	**H**LAESHANK	HNE + 158.1	1253.65	b ions: **296.20** (**b1**), **409.28** (**b2**), **480.32** (**b3**), **609.74** (**b4**), **696.82** (**b5**), **833.96** (**b6**), **905.04** (**b7**), **1019.15** (**b8**) y ions: 145.10 (y1), 259.14 (y2), 330.18 (y3), 467.24 (y4), 554.27 (y5), 683.70 (y6), 754.78 (y7), 867.94 (y8)
88–96	HLAESHANK	Unadducted	1271.66	b ions: 138.07 (b1), 251.15 (b2), 322.19 (b3), 451.23 (b4), 538.26 (b5), 675.32 (b6), 746.36 (b7), 860.40 (b8) y ions: 145.10 (y1), 259.14 (y2), 330.18 (y3), 467.24 (y4), 554.27 (y5), 683.70 (y6), 754.78 (y7), 867.94 (y8)
88–96	HLAES**H**ANK	HNE + 158.1	1271.66	b ions: 138.07 (b1), 251.15 (b2), 322.19 (b3), 451.23 (b4), 538.26 (b5), **833.96** (**b6**), **905.04** (**b7**), **1019.15** (**b8**) y ions: 145.10 (y1), 259.14 (y2), 330.18 (y3), **625.75** (**y4**), **712.82** (**y5**), **841.94** (**y6**), **913.02** (**y7**), **1026.18** (**y8**)
119–133	HPSDFGADAQAAMSK	Unadducted	1514.67	b ions: 138.07 (b1), 235.12 (b2), 322.15 (b3), 437.18 (b4), 584.25 (b5), 641.27 (b6), 712.31 (b7), 827.33 (b8), 898.37 (b9), 1026.43 (b10), 1097.47 (b11), 1168.50 (b12) y ions: 363.17 (y1), 434.21 (y2), 505.24 (y3), 633.30 (y4), 704.34 (y5), 819.37 (y6), 890.40 (y7), 947.43 (y8), 1094.49 (y9), 1209.52 (y10), 1296.55 (y11), 1393.61 (y12)
119–133	**H**PSDFGADAQAAMSK	HNE + 158.1	1532.68	b ions: **296.20** (**b1**), **393.25** (**b2**), **480.28** (**b3**), **595.67** (**b4**), **742.85** (**b5**), **799.90** (**b6**), **870.98** (**b7**), **986.07** (**b8**), **1057.15** (**b9**), **1185.28** (**b10**), **1256.36** (**b11**), **1327.44** (**b12**) y ions: 363.17 (y1), 434.21 (y2), 505.24 (y3), 633.75 (y4), 704.83 (y5), 819.91 (y6), 890.99 (y7), 948.05 (y8), 1095.22 (y9), 1210.31 (y10), 1297.39 (y11), 1394.51 (y12)
148–153	VLGFHG	Unadducted	629.34	b ions: 100.08 (b1), 213.16 (b2), 270.18 (b3), 417.25 (b4), 554.31 (b5) y ions: 74.02 (y1), 211.08 (y2), 358.15 (y3), 415.17 (y4), 528.26 (y5)
148–153	VLGF**H**G	HNE + 158.1	629.34	b ions: 100.08 (b1), 213.16 (b2), 270.18 (b3), 417.25 (b4), **712.91** (**b5**) y ions: 74.02 (y1), **369.21** (**y2**), **516.28** (**y3**), **573.30** (**y4**), **686.83** (**y5**)

a) Amino acid position in the water buffalo Mb; b) Amino acid sequence in the water buffalo Mb; c) Observed signals assigned as *b* or *y* ions. Ions showing shift in the mass by 156 Da indicating HNE adduction with respect to corresponding ions in unmodified peptides are highlighted in bold fonts

**Table 3:** MS/MS spectral features of unadducted and HNE-adducted goat (*Capra hircus*) meat myoglobin peptides

Peptide position^a)^	Peptide sequence^b)^	Modification and mass shift	Precursor *m*/*z*	b and y ions identified^c)^
17–31	VEADVAGHGQEVLIR	Unadducted	1592.77	b ions: 100.08 (b1), 229.12 (b2), 300.16 (b3), 415.16 (b4), 514.25 (b5), 585.29 (b6), 642.31 (b7), 779.37 (b8), 836.39 (b9), 964.45 (b10), 1093.49 (b11), 1192.56 (b12), 1305.64 (b13), 1418.73 (b14) y ions: 174.11 (y1), 287.20 (y2), 400.28 (y3), 499.35 (y4), 628.39 (y5), 756.45 (y6), 813.47 (y7), 950.53 (y8), 1107.55 (y9), 1078.59 (y10), 1177.66 (y11), 1292.68 (y12), 1363.72 (y13), 1492.76 (y14)
17–31	VEADVAG**H**GQEVLIR	HNE + 158.1	1751.01	b ions: 100.08 (b1), 229.12 (b2), 300.16 (b3), 415.16 (b4), 514.25 (b5), 585.29 (b6), 642.31 (b7), **937.50** (**b8**), **994.52** (**b9**), **1122.58** (**b10**), **1251.62** (**b11**), **1350.69** (**b12**), **1463.77** (**b13**), **1576.86** (**b14**) y ions: 174.11 (y1), 287.20 (y2), 400.28 (y3), 499.35 (y4), 628.39 (y5), 756.45 (y6), 813.47 (y7), **1108.66** (**y8**), **1165.68** (**y9**), **1236.72** (**y10**), **1335.79** (**y11**), **1450.81** (**y12**), **1521.85** (**y13**), **1650.89** (**y14**)
32–42	LFTGHPETLEK	Unadducted	1271.43	b ions: 114.09 (b1), 261.16 (b2), 362.21 (b3), 419.23 (b4), 556.29 (b5), 653.34 (b6), 782.38 (b7), 883.43 (b8), 996.52 (b9), 1125.56 (b10) y ions: 146.11 (y1), 275.15 (y2), 388.23 (y3), 489.28 (y4), 618.32 (y5), 715.38 (y6), 852.43 (y7), 909.46 (y8), 1010.50 (y9), 1157.57 (y10)
32–42	LFTG**H**PETLEK	HNE + 158.1	1429.67	b ions: 114.09 (b1), 261.16 (b2), 362.21 (b3), 419.23 (b4), **714.42** (**b5**), **811.47** (**b6**), **940.51** (**b7**), **1041.56** (**b8**), **1154.65** (**b9**), **1283.69** (**b10**) y ions: 146.11 (y1), 275.15 (y2), 388.23 (y3), 489.28 (y4), 618.32 (y5), 715.38 (y6), **1010.56** (**y7**), **1067.59** (**y8**), **1168.63** (**y9**), **1315.70** (**y10**)
63–77	KHGNTVLTALGGILK	Unadducted	1521.82	b ions: 129.10 (b1), 266.16 (b2), 323.18 (b3), 437.23 (b4), 538.27 (b5), 637.34 (b6), 750.43 (b7), 851.47 (b8), 922.51 (b9), 1035.60 (b10), 1092.62 (b11), 1149.64 (b12), 1262.72 (b13), 1375.81 (b14) y ions: 146.11 (y1), 259.19 (y2), 372.27 (y3), 429.30 (y4), 486.32 (y5), 599.40 (y6), 670.44 (y7), 771.49 (y8), 884.57 (y9), 983.64 (y10), 1084.69 (y11), 1198.73 (y12), 1255.75 (y13), 1392.81 (y14)
63–77	K**H**GNTVLTALGGILK	HNE + 158.1	1680.06	b ions: 129.10 (b1), **424.29** (**b2**), **481.31** (**b3**), **595.36** (**b4**), **696.40** (**b5**), **795.47** (**b6**), **908.56** (**b7**), **1009.60** (**b8**), **1080.64** (**b9**), **1193.73** (**b10**), **1250.75** (**b11**), **1307.77** (**b12**), **1420.85** (**b13**), **1533.94** (**b14**) y ions: 146.11 (y1), 259.11 (y2), 372.27 (y3), 429.30 (y4), 486.32 (y5), 599.40 (y6), 670.44 (y7), 771.49 (y8), 884.57 (y9), 983.64 (y10), 1084.69 (y11), 1198.73 (y12), 1255.75 (y13), **1550.94** (**y14**)
80–87	GHHEAEVK	Unadducted	905.96	b ions: 58.03 (b1), 195.09 (b2), 332.15 (b3), 461.19 (b4), 532.23 (b5), 661.27 (b6), 760.34 (b7) y ions: 146.11 (y1), 245.17 (y2), 374.22 (y3), 445.25 (y4), 574.30 (y5), 711.36 (y6), 848.41 (y7)
80–87	G**H**HEAEVK	HNE + 158.1	1064.20	b ions: 58.03 (b1), **353.22** (**b2**), **490.28** (**b3**), **619.32** (**b4**), **690.36** (**b5**), **819.40** (**b6**), **918.47** (**b7**) y ions: 146.11 (y1), 245.17 (y2), 374.22 (y3), 445.25 (y4), 574.30 (y5), 711.36 (y6), **1006.54** (**y7**)
88–96	HLAESHANK	Unadducted	1271.40	b ions: 138.07 (b1), 251.15 (b2), 322.19 (b3), 451.23 (b4), 538.26 (b5), 675.32 (b6), 746.36 (b7), 860.40 (b8), 988.50 (b9), 1125.56 (b10) y ions: 146.11 (y1), 283.16 (y2), 411.26 (y3), 525.30 (y4), 596.34 (y5), 733.40 (y6), 820.43 (y7), 949.47 (y8), 1020.51 (y9), 1133.59 (y10)
88–96	**H**LAESHANK	HNE + 158.1	1164.32	b ions: **296.20** (**b1**), **409.28** (**b2**), **480.38** (**b3**), **609.36** (**b4**), **696.39** (**b5**), **833.45** (**b6**), **904.49** (**b7**), **1018.53** (**b8**) y ions: 146.11 (y1), 260.15 (y2), 331.19 (y3), 468.24 (y4), 555.28 (y5), 684.32 (y6), 755.36 (y7), 868.44 (y8)
88–98	HLAESHANKHK	Unadducted	1271.40	b ions: 138.07 (b1), 251.15 (b2), 322.19 (b3), 451.23 (b4), 538.26 (b5), 675.32 (b6), 746.36 (b7), 860.40 (b8), 988.50 (b9), 1125.56 (b10) y ions: 146.11 (y1), 283.16 (y2), 411.26 (y3), 525.30 (y4), 596.34 (y5), 733.40 (y6), 820.43 (y7), 949.47 (y8), 1020.51 (y9), 1133.59 (y10)
88–98	HLAESHANK**H**K	HNE + 158.1	1429.64	b ions:138.07 (b1), 251.15 (b2), 322.19 (b3), 451.23 (b4), 538.26 (b5), 675.32 (b6), 746.36 (b7), 860.40 (b8), 988.50 (b9), **1283.69** (**b10**) y ions: 146.11 (y1), **441.30** (**y2**), **569.39** (**y3**), **683.43** (**y4**), **754.47** (**y5**), **891.53** (**y6**), **978.56** (**y7**), **1107.60** (**y8**), **1178.64** (**y9**), **1291.72** (**y10**)
103–118	YLEFISDAIIHVLHAK	Unadducted	1869.19	b ions: 164.07 (b1), 277.16 (b2), 406.20 (b3), 553.27 (b4), 666.35 (b5), 753.38 (b6), 868.41 (b7), 939.45 (b8), 1052.53 (b9), 1165.61 (b10), 1302.67 (b11), 1401.74 (b12), 1514.83 (b13), 1651.88 (b14), 1722.92 (b15) y ions: 146.11 (y1), 217.14 (y2), 354.20 (y3), 467.29 (y4), 566.35 (y5), 703.41 (y6), 816.50 (y7), 929.58 (y8), 1000.62 (y9), 1115.65 (y10), 1202.68 (y11), 1315.76 (y12), 1462.83 (y13), 1591.95 (y14), 1704.96 (y15)
103–118	YLEFISDAII**H**VLHAK	HNE + 158.1	2027.43	b ions: 164.07 (b1), 277.16 (b2), 406.20 (b3), 553.27 (b4), 666.35 (b5), 753.38 (b6), 868.41 (b7), 939.45 (b8), 1052.53 (b9), 1165.61 (b10), **1460.80** (**b11**), **1559.87** (**b12**), **1672.96** (**b13**), **1810.02** (**b14**), **1881.05** (**b15**) y ions: 146.11 (y1), 217.14 (y2), 354.20 (y3), 467.29 (y4), 566.35 (y5), **861.54** (**y6**), **974.63** (**y7**), **1087.71** (**y8**), **1158.75** (**y9**), **1273.78** (**y10**), **1360.81** (**y11**), **1473.89** (**y12**), **1620.96** (**y13**), **1750.00** (**y14**), **1863.09** (**y15**)
103–118	YLEFISDAIIHVLHAK	Unadducted	1869.19	b ions: 164.07 (b1), 277.16 (b2), 406.20 (b3), 553.27 (b4), 666.35 (b5), 753.38 (b6), 868.41 (b7), 939.45 (b8), 1052.53 (b9), 1165.61 (b10), 1302.67 (b11), 1401.74 (b12), 1514.83 (b13), 1651.88 (b14), 1722.92 (b15) y ions: 146.11 (y1), 217.14 (y2), 354.20 (y3), 467.29 (y4), 566.35 (y5), 703.41 (y6), 816.50 (y7), 929.58 (y8), 1000.62 (y9), 1115.65 (y10), 1202.68 (y11), 1315.76 (y12), 1462.83 (y13), 1591.95 (y14), 1704.96 (y15)
103–118	YLEFISDAIIHVL**H**AK	HNE + 158.1	2027.43	b ions: 164.07 (b1), 277.16 (b2), 406.20 (b3), 553.27 (b4), 666.35 (b5), 753.38 (b6), 868.41 (b7), 939.45 (b8), 1052.53 (b9), 1165.61 (b10), 1302.67 (b11), 1401.74 (b12), 1514.83 (b13), **1810.02** (**b14**), **1881.05** (**b15**) y ions: 146.11 (y1), 217.14 (y2), **512.33** (**y3**), **625.42** (**y4**), **724.48** (**y5**), **861.54** (**y6**), **974.63** (**y7**), **1087.71** (**y8**), **1158.75** (**y9**), **1273.78** (**y10**), **1360.81** (**y11**), **1473.89** (**y12**), **1620.96** (**y13**), **1750.00** (**y14**), **1863.09** (**y15**)
119–133	HPSDFGADAQGAMSK	Unadducted	1518.62	b ions: 138.07 (b1), 235.12 (b2), 322.15 (b3), 437.18 (b4), 584.25 (b5), 641.27 (b6), 712.31 (b7), 827.33 (b8), 898.37 (b9), 1026.43 (b10), 1083.45 (b11), 1154.49 (b12), 1285.53 (b13), 1372.56 (b14) y ions: 146.11 (y1), 233.14 (y2), 364.18 (y3), 435.22 (y4), 492.4 (y5), 620.30 (y6), 691.33 (y7), 806.36 (y8), 877.40 (y9), 934.42 (y10), 1081.49 (y11), 1196.51 (y12), 1283.55 (y13), 1380.60 (y14)
119–133	**H**PSDFGADAQGAMSK	HNE + 158.1	1676.86	b ions: **296.20** (**b1**), **393.25** (**b2**), **480.28** (**b3**), **595.67** (**b4**), **742.85** (**b5**), **799.40** (**b6**), **870.44** (**b7**), **985.46** (**b8**), **1056.50** (**b9**), **1184.56** (**b10**), **1241.58** (**b11**), **1312.62** (**b12**), **1443.66** (**b13**), **1530.69** (**b14**) y ions: 146.11 (y1), 233.14 (y2), 364.18 (y3), 435.22 (y4), 492.24 (y5), 620.30 (y6), 691.33 (y7), 806.36 (y8), 877.40 (y9), 934.42 (y10), 1081.49 (y11), 1196.51 (y12), 1283.55 (y13), 1380.60 (y14)

a) Amino acid position in the goat Mb; b) Amino acid sequence in the goat Mb; c) Observed signals assigned as *b* or *y* ions. Ions showing shift in the mass by 156 Da indicating HNE adduction with respect to corresponding ions in unmodified peptides are highlighted in bold fonts

An interesting finding in our study is that, goat Mb with 95.4% and 97.4% sequence identity with buffalo and beef Mb, respectively with 12 total histidine residues exhibited 9 HNE adductions. Similar to buffalo Mb, even for goat Mb HIS 113 and HIS 116 originating from same peptide (YLEFISDAIIHVLHAK, position 103–118) were found adducted to HNE. The difference in HNE adductions between buffalo and goat Mb’s is presented in Table 4. These results indicate a difference in HNE adduction between buffalo and goat Mb’s both in terms of number and location of adductions. We observed the adduction of proximal HIS 93 in buffalo, whereas for goat the adduction of distal HIS 64 was found. This is of great significance as HIS 93, the proximal histidine, is bound to the heme moiety of Mb, whereas distal HIS 64, coordinates with oxygen or other molecules associated with the sixth ligand during the interconversion of Mb redox forms (i.e., Oxy-Mb and Met-Mb). Because HIS 93 and 64 lie in close proximity to the heme group, their modification by HNE could be expected to alter the protein structure around the heme cleft and subsequently impact redox stability. In their study comparing HNE induced oxidation of beef and pork Oxy-Mb’s Suman et al. (2007) observed that fewer HIS residues were adducted by HNE in porcine Mb when compared to bovine Mb, which suggested an apparently lower susceptibility of porcine Mb to the redox destabilizing effect of HNE [12]. They concluded that preferential HNE adduction at proximal residue (HIS 93), exclusively observed in bovine Oxy-Mb might result in more pronounced Oxy-Mb oxidation in beef relative to pork. Our findings indicate that goat Oxy-Mb with HNE adduction at distal histidine (HIS 64) results in more pronounced Oxy-Mb oxidation resulting in higher % Met-Mb than beef Mb with HNE adduction at proximal histidine (HIS 93). Yin et al. (2011) studied the HNE induced oxidation of Oxy-Mb’s from seven different meat animals and concluded that, the relative effect of HNE was greater for Mb’s that contained 12 ± 1 histidine residues than for those that contained nine histidine residues [31]. Table 4 clearly indicate greater number of adductions and alkylation of more HIS residues in red meat producing livestocks (12 or 13 HIS) except pork (nine histidine) relative to poultry birds (eight or nine histidine). Present study confirm these findings, wherein livestock Mb’s with higher number of histidine residues results in greater Mb-HNE adducts and more covalently modified histidine residues leading to increased susceptibility for lipid oxidation induced oxidation.

**Table 4:** Comparison of Mb-HNE adductions and number of covalently modified histidine (HIS) residues in different meat animals and poultry

Meat animals and poultry	Total No. HIS residues	Mb-HNE incubation conditions	Incubation time	No. Mb-HNE adductions	Modified HIS residues	Position of adducted HIS residues	Reference
Meat animals							
Water buffalo (bovine)	13	pH 7.4, 37 °C	210 min	3	7	24,36,81,88,93,119,152	Present study
Goat (caprine)	12	pH 7.4, 37 °C	210 min	5	9	24,36,64,81,88,97,113, 116,119	Present study
Horse (equine)	11	pH 7.4, 37 °C	160 min	3	--------	--------	[9]
Cattle (bovine)	13	pH 7.4, 37 °C	160 min	3	7	24,36,81,88,93,119,152	[12]
				3	6	24,64,93,116,119,152	[10]
Pig (porcine)	9	pH 7.4, 37 °C	240 min	1	3	24,36,119	[12]
Sheep (ovine)	12	pH 7.4, 37 °C	240 min	3	3	25,65,120	[18]
Poultry							
Chicken	9	pH 5.8, 25 °C	240 min	2	2	64,93	[11]
Turkey	9	pH 5.8, 25 °C	240 min	2	--------	--------	[11]
Ostrich	8	pH 7.4, 37 °C	360 min	1	1	36	[17]
Emu	9	pH 7.4, 37 °C	360 min	1	2	34,36	[17]

## Conclusions

In conclusion, results obtained from this study revealed that buffalo and goat Mb’s were similar in behaviour during isolation and purification and exhibited a molecular mass of 17,043.6 Daltons and 16,899 Daltons, respectively. The study has demonstrated the species-specific variation in 2DE properties of buffalo and goat Mb’s. Present study demonstrated the suitability of OFFGEL electrophoresis for determining the p*I* of Mb proteins. The study also reinforces the potential interaction between water buffalo and goat Mb’s with a lipid derived aldehyde (HNE) via covalent modification of seven and nine histidine residues, respectively. The results suggest lower susceptibility of water buffalo Mb to the redox destabilizing effect of HNE compared to goat Mb. Our findings provide explanation for the previously noted observation that more number of Mb-HIS adducts and increased alkylation of HIS residues will exacerbate the Oxy-Mb oxidation.

## Methods

### Materials

Sephacryl S-200 HR and sodium hydrosulfite were obtained from Sigma-Aldrich chemicals Co., Sweden. The disposable PD-10 columns, IPG strips, DTT, Iodoacetamide, Acrylamide, Bis-acrylamide, Glycine, Methanol, Glacial Acetic acid, β-mercaptoenthanol and Coomassie blue, were obtained from GE Health Care. HNE was obtained from Cayman Chemical Company. Dialysis tubing was sourced from Spectrum laboratories, Inc. (Rancho Domingues, CA, USA). OFFGEL starter kit was procured from Agilent Technologies. All chemicals were of reagent grade or greater purity.

### Isolation and purification of buffalo and goat myoglobins

Buffalo and goat hearts procured from municipal abattoir, Chengicherla, Hyderabad, India were trimmed off fat and connective tissue, vacuum packaged and stored at−80 °C till extraction. Myoglobin (Mb) was isolated from cardiac muscles [22]. Frozen cardiac muscle samples were chilled overnight in refrigerator, cut into small pieces and homogenized with buffer containing 10 mM Tris–HCl with 1 mM EDTA (pH 8.0) and centrifuged at 5000 g for 10 min at 4 °C. The supernatant was brought to 70% ammonium sulfate saturation and centrifuged at 18000 g for 20 min 4 °C. The resulting supernatant was brought to 100% saturation with ammonium sulfate and centrifuged at 20000 g for 1 h 4 °C. The precipitate was resuspended in homogenization buffer and dialyzed against ten volumes of dialysis buffer (5 mM Tris–HCl with 1 mM EDTA, pH 8.0) for 24 h, with buffer changes at every 8 h interval. The dialysate was filtered through 0.45 μm syringe filter followed by and 0.22 μm filter. The 3 mL filtrate with a protein concentration of approximately 10 mg/mL was loaded on to Sephacryl S-200 HR gel filtration column (Econo column, 1.5 × 100 cm, BIORAD) equilibrated with elution buffer (5 mM Tris–HCl with 1 mM EDTA, pH 8.0) at a flow rate of 0.1 mL/min. Purified Mb fractions (1 mL) were collected and absorbance was measured at 280 and 540 nm using UV–VIS spectrophotometer (Model: UV-1700, PharmaSpec, SHIMADZU, Japan). To confirm the purity of Mb, fractions were subjected to sodium dodecyl sulphate-polyacrylamide gel electrophoresis (SDS-PAGE) and 2-dimensional gel electrophoresis (2DE).

### Isolation of sarcoplasmic proteome

The sarcoplasmic proteome from buffalo and goat samples were extracted as described by Sentandreu et al. (2010) [32]. Frozen samples (1 g) were cut and homogenized in 10 mL cold extraction buffer (50 mM Tris, and pH 8.0). The homogenate was centrifuged at 10,000 g for 20 min at 4 °C. The supernatant constituting the sarcoplasmic extract in which all soluble proteins were contained was filtered and used for 2DE.

### Sodium dodecyl sulfate polyacrylamide gel electrophoresis

SDS-PAGE analyses of buffalo and goat Mb fractions from different steps of purification were performed using the method of Laemmli (1970) under reducing conditions using a 4% stacking gel and 12% separating gel in midi-electrophoresis apparatus (Model: SE-600 Ruby; GE Healthcare, Uppsala, Sweeden) [33]. The current for each gel was maintained at 10 mA. After separation, the proteins were stained with 0.1% (w/v) coomassie brilliant blue R-250 in 50% (v/v) methanol and 10% (v/v) acetic acid, and destained with 40% methanol (v/v) and 10% (v/v) acetic acid. Destained images were scanned using Image Scanner-III, LabScan 6.0 (GE Healthcare, Uppsala, Sweeden) and IQTL calibration converter was used to obtain image.

### Two-dimensional gel electrophoresis and image analysis

Sarcoplasmic extract and gel-filtered (pure) Mb samples were passed through PD-10 desalting columns equilibrated with double distilled water to remove the salts. Later, protein concentration was determined using 2-D Quant Kit (GE Healthcare, USA) and 2DE was performed as described by Lametsch and Benedixen (2001) with few modifications [34]. The immobilized pH gradient (IPG) strips (Immobiline®drystrip, 11 cm, pH range 3–10) were passively rehydrated in 200 μL of myoglobin samples containing 750–800 μg proteins for 12 h. Rehydrated IPG strips were subjected to Iso-electric focusing (IEF) in a Ettan IPGPhor-3 (GE health care, Uppsala, Sweeden) gel apparatus at 18 °C for a total of 10800 Vh. Focused IPG strips were equilibrated in 6 M urea, 20% glycerol, 2% SDS, 0.375 M Tris (pH 8.8), for 15 min in 2% dithiothreitol (DTT) and 5% iodoacetamide respectively. After equilibration, proteins were separated in the second dimension, with the SE 600 Ruby apparatus at 100 V with 60 mA/gel until tracking dye reached lower end of gel. The gel was removed and stained with colloidal Coomassie blue for 3 h followed by overnight destaining. Myoglobin samples from buffalo and goat were run under the same conditions and the three gels were produced for each.

Stained gels were scanned on an Image Scanner III using labscan 6.0 software. Spot detection and quantification were performed with Image Master Platinum7.0 software (GE Healthcare, Uppsala, Sweeden). The images were grouped into different groups according to required study and the intensity and area of individual spots were analyzed for comparative image analysis. For each spot in buffalo or goat Mb samples, spot quantity values in triplicate gels were averaged for statistical analysis. A spot was considered to be significant in differential abundance when it was associated with *P* < 0.05.

### Determination of molecular mass using MALDI-TOF MS and protein identification through peptide mass fingerprinting (PMF)

Purified Mb was mixed 1:1 with 50% sinapinic acid and the mixture was allowed to crystallize on the MALDI target plate for 10 min. Protein molecular ions were analyzed in linear, positive ion mode using MALDI-TOF ULTRAFLEX III instrument (MALDI-TOF MS Bruker Daltonics, ULTRAFLEX III), using an acceleration voltage of 2.2 kV. The instrument was calibrated using lysozyme and beta-lactoglobulin as protein standards. The resulting spectra (from 1000 laser shots) were averaged, noise-smoothed, baseline-corrected, and analysed [12].

In-gel digestion of the myoglobin protein separated by 2DE was performed as described by Shevchenko et al. (2006) with slight modifications [35]. The PMF measured mass-to-charge ratio (*m*/*z*) values of peptides resulting from a protein digest form the basis for a characteristic fingerprint of the intact protein. Desalted peptide extracts (0.3 μL) were spotted onto MALDI target plate with 0.3 μL of 5 mg/mL α-cyano-4-hydroxycinnamic acid (Aldrich, St. Louis, MO, USA) in 50% CH_3_CN/50% 0.1% trifluoroacetic acid. Crystallized samples were washed with cold 0.1% trifluoroacetic acid and were analyzed in linear, positive ion mode on the MALDI-TOF ULTRAFLEX III instrument (MALDI-TOF MS Bruker Daltonics ULTRAFLEX III). The resulting spectra (from 600 laser shots) were averaged; noise-smoothed, baseline-corrected and further analysis was done with FLEX ANALYSIS SOFTWARE for obtaining the peptide mass fingerprint [36]. The masses obtained in the peptide mass fingerprint were submitted for MASCOT 2.2 search in NCBI database for identification of the protein.

### Determination of isoelectric point using OFFGEL electrophoresis

Buffalo and goat Mb protein samples were fractionated by isoelectric focusing using an Agilent 3100 OFFGEL Fractionator (Agilent Technologies, Santa Clara, CA, USA), following manufacturer’s instructions. Parts were assembled, Immobilised pH gradient (IPG) strips were rehydrated, and protein samples were diluted into the 1.25× OFFGEL stock solution as instructed in the Agilent Quick Start Guide. Mb proteins were focused based on their p*I* using two different 24 cm long IPG strips with a linear pH gradient ranging from 3.0 to 10.0 placed in the same tray following the Agilent method. A total of 0.5 mg of Mb protein was loaded onto each IPG strip. At the end of the run, samples from each well were directly collected and subjected to SDS-PAGE analysis.

### Reaction of buffalo and goat OxyMb’s with HNE

Buffalo (0.15 mM) and goat (0.075 mM) Oxy-Mb’s were each combined with 1.0 and 0.5 mM of 4-Hydroxy-2-nonenal, respectively, at 37 °C, pH 7.4 and 25 °C, pH 5.6, the typical pH in live animals and post-mortem muscles. These Mb concentrations were selected according to our earlier studies as they reflect the average Mb levels found in buffalo (5.0 mg Mb/g tissue) and goat (2.5 mg Mb/g tissue) meats. The molar ratio of Mb: aldehydes was maintained at 1:7 for all reactions [9]. Controls consisted of Oxy-Mb and a volume of ethanol equivalent to that used to deliver the HNE to the treatment mixture. During incubation, the control (Oxy-Mb + ethanol) and treatment (Oxy-Mb + HNE) solutions were scanned spectrophotometrically from 650 to 450 nm using a UV–VIS spectrophotometer (Model: UV-1700, PharmaSpec, SHIMADZU, Japan). The blank contained only buffer (10 mM Tris–HCl with 1 mM EDTA). Metmyoglobin formation was calculated using absorbance values at 503, 557, and 582 nm [37]. At the end of incubation, Mb-HNE mix was directly submitted for MALDI-TOF analysis, whereas for ESI-QTOF MS/MS, the mix was subjected to SDS-PAGE and the gel bands were excised, digested and analysed.

### MALDI-TOF MS and ESI-QTOF MS/MS

Native and HNE-treated buffalo and goat myoglobins (intact proteins) were analyzed by MALDI-TOF MS to detect changes in the total mass resulting from potential HNE adduction. One mL of the reaction solution was passed over a PD-10 desalting column calibrated with distilled water to remove unreacted 4-hydroxynonenal. Buffalo and goat Mb-HNE adducts were detected using MALDI-TOF MS as explained earlier.

SDS–PAGE of native and HNE-treated buffalo and goat Mb’s (after 3 h at 37 °C and pH 7.4) was performed under reducing conditions using a midi-gel electrophoresis unit. Each band of native and HNE-treated buffalo and goat Mb’s on Coomassie-stained gels were excised, destained and digested [35] with sequencing-grade trypsin at 37 °C for 18 h . After digestion, the sample was vacuum dried and was dissolved in 50 μl of 0.1% Formic acid. One μl of each of the samples were separated on the Nano-Acquity BEH C18 column (100 μm i.d. × 100 mm) connected to WATERS nanoUPLC system for 150 min with 50% gradient of water 0.1%formic acid (buffer A) and ACN, 0.1% formic acid at a flow rate of 400 nL/min. The nano LC separated peptides were analysed for MS and MS/MS fragmentation on SYNAPT G2 WATERS nLC coupled QTOF mass spectrometer with ESI source.

The MS/MS spectra were acquired in data-dependent scanning mode with one full MS scan with a *m*/*z* of 50–1000 followed by four MS/MS scans on the first four most intense ions with dynamic exclusion of previously selected precursors for a period of 1 min. MS/MS spectra were matched to Mb database sequences using the protein database search software MASCOT search engine (http://www.matrixscience.com) with a parent ion tolerance of 2.5 amu and fragment ion tolerance of 1 Da. Protein identification was done using the WATERS Protein Lynx Global Server (PLGS) v4.1 against the NCBI or UNIPROT database of *Bubbalis bubbalis* and *Capra hircus* Myoglobin. The raw data was obtained for the samples and was analysed in MASSLYNX (v4.1) using the BIOLYNX Tool and were checked for HNE modifications in the HISTIDINE sites of the respective myoglobin. The option Chordata (vertebrates and relatives) was selected as taxonomy restriction parameter. Electrophile adduction in several proteins has been identified successfully utilizing SALSA (Scoring Algorithm for Spectral Analysis).

### Statistical analysis

Statistical analysis was performed with the analysis of variance (ANOVA) using SPSS (SPSS version 13.0 for windows; SPSS, Chicago, IL, USA). Least square means for F-tests were calculated by using Duncan’s multiple range tests and were considered significant at *p* < 0.05. The experimental design for evaluation of Mb oxidation study was a completely randomized design with three replicates (*n* = 3). The data for buffalo and goat were analyzed separately using two-way analysis of variance (ANOVA) option in SPSS and the differences among means were detected using the least significance difference (LSD) at a 5% level.

## Abbreviations

2DE: Two-dimensional gel electrophoresis
ESI-QTOF MS/MS: Electrospray ionization quadrupole time-of-flight tandem mass spectrometry
HNE: 4-Hydroxy-2-nonenal
IPG: Immobilized pH gradient
*m*/*z*: Mass-to-charge ratio
MALDI-TOF-MS: Matrix-assisted laser desorption ionization-time of flight mass spectrometry
Mb: Myoglobin
MS: Mass spectrometry
OxyMb: Oxymyoglobin
PMF: Peptide mass fingerprinting
SDS-PAGE: Sodium dodecyl sulfate polyacrylamide gel electrophoresis
